# Strategies to overcome trastuzumab resistance in HER2-overexpressing breast cancers: focus on new data from clinical trials

**DOI:** 10.1186/s12916-014-0132-3

**Published:** 2014-08-12

**Authors:** Pernelle Lavaud, Fabrice Andre

**Affiliations:** Department of Medical Oncology, Gustave Roussy Cancer Center, Villejuif, France; Université Paris Sud, Faculté de Médecine, Kremlin Bicêtre, France

**Keywords:** Breast cancer, HER-2, Resistance, Tyrosine kinase inhibitors, Monoclonal antibodies

## Abstract

Breast cancers over-express the human epidermal growth factor receptor 2 (HER2) in about 15% of patients. This transmembrane tyrosine kinase receptor activates downstream signaling pathways and leads to proliferation of cancer cells. Trastuzumab, an anti-HER2 monoclonal antibody, improves outcome in women with early and metastatic breast cancer. Resistance to trastuzumab involves the phosphoinositide 3-kinase/mammalian target of rapamycin (PI3K/mTOR) pathway, truncation of the Her2 receptor or lack of immune response. The last decade has seen major advances in strategies to overcome resistance to trastuzumab. This includes the development of antibody-drug conjugates, dual HER2 inhibition strategies, inhibition of PI3K/mTOR pathway and development of modulators of immune checkpoints.

## Introduction

About 15% of breast cancer over-expresses the human epidermal growth factor receptor 2 (HER2) [1,2]. HER2 is a transmembrane tyrosine kinase receptor that is a member of the human epidermal growth factor receptor (EGFR/HER) family. This receptor is involved in proliferation and survival of epithelial cells. Activation of HER2 generates activation of downstream signaling pathways including mitogen-activated protein kinase (MAPK) and phosphoinositide 3-kinase (PI3K/Akt) [3]. Over-expression of HER2 predicts poorer prognosis [4,5] and higher sensitivity to chemotherapy, such as anthracycline and paclitaxel [5,6]. On the opposite side, HER2 overexpression has been associated with resistance to tamoxifen [6–9]. Finally, HER2 positive breast cancer is associated with an increased risk of brain metastases [10].

Trastuzumab is a humanized recombinant monoclonal antibody that targets the HER2 extracellular domain. The use of trastuzumab is considered as standard of care both in early and metastatic HER2 over-expressing breast cancer. Numerous clinical trials have confirmed that trastuzumab improves overall survival (OS) in metastatic breast cancers [11–13]. In early breast cancers, it improves disease free survival (DFS) and OS [14–16] and increases pathological complete response (pCR) in a neoadjuvant setting when combined with chemotherapy (66.7% with trastuzumab versus 25% without) [14,17,18].

Its antitumor activity is hypothesized to be related to two different mechanisms of action: downregulation of the intracellular signaling pathway *via* the PI3K and MAPK pathways, and activation of the immune response via antibody dependent cell-mediated cytotoxicity (ADCC) and eventually adaptive immune response [19–22].

Unfortunately, resistances to trastuzumab occur, mainly in the metastatic setting, where most of the patients treated with trastuzumab have a disease progression within one year [23]. Molecular mechanisms of trastuzumab resistance may involve signaling from other HER receptors, such as HER3 or epidermal growth factor receptor (EGFR) [24], insulin-like growth factor receptor [25,26], activation of PI3K/AKT/mTOR [27], overexpression of c-MET [28] or loss of PTEN (phosphatase and TENsin homolog) [27,29], up-regulation of src activity [30] or MUC4 [31,32], increased VEGF (vascular endothelial growth factor) expression [33], expression of the p95 isoform of HER2 [34] and co-expression of EGFR [35].

**Figure 1:**
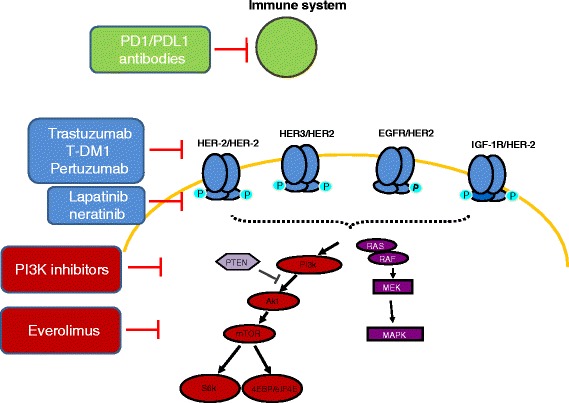
**HER2-directed drugs and targets; mechanisms of action.**

In the present review, we will present clinical data on the main strategies that aimed at overcoming trastuzumab resistance. The targets and drug family under investigation are reported in Figure 1. The results of the main randomized trials are summarized in Table 1.

**Table 1:** **Summary of randomized trials testing new drugs in Her2-overexpressing breast cancers**

**Drugs**	**Targets**	**Study**	**Identifier number**	**Phase**	**Setting**	**Primary endpoint**	**Therapy**	**Results**	
Pertuzumab	HER2/3	CLEOPATRA	NCT00567190	Phase III	MBC/locally recurent/unresectable	PFS	Trastuzumab + docetaxel + pertuzumab	18.7 months	HR 0.69; 95% CI 0.58-0.81; *P* <0.001
					in first line		trastuzumab + docetaxel + placebo	12.4 months	
		NEOSPHERE	NCT00545688	Phase II	Neo adjuvant	pCR	Trastuzumab + docetaxel	29.0%	
							Trastuzumab + pertuzumab + docetaxel	45.8%	*P* = 0.0141
							Pertuzumab + docetaxel	24.0%	
							Trastuzumab + pertuzumab	16.8%	
T-DM1	HER2	EMILIA	NCT00829166	Phase III	MBC	PFS	T-DM1	9.6 months	HR 0.65; 95% CI 0.55-0.77; *P* <0.001
							Lapatinib + Capecitabine	6.4 months	
		TH3RESA	NCT01419197	Phase III	MBC (in third line)		T-DM1	6.2 months	HR 0.528; 95% CI 0.422-0.661; *P* <0.0001
							Physician’s choice treatment	3.3 months	
Lapatinib	HER1/HER2	EGF104900	NCT00320385	Phase III	MBC trastuzumab resistant	PFS	Lapatinib + trastuzumab Lapatinib	11.1 months	HR 0.74; 95% CI 0.58-0.94; *P* = 0.011
								8.1 months	
		NSABP B-41	NCT00486668	Phase III	Neo adjuvant	pCR	Paclitaxel + Trastuzumab	53.5%	
							Paclitaxel + Lapatinb	52.5%	
							Paclitaxel + Lapatinib + Trastuzumab	62.0%	
		NeoALTTO	NCT00553358	Phase III	Neo adjuvant	pCR	Paclitaxel + Trastuzumab	29.5%	
							Paclitaxel + Lapatinb	24.7%	
							Paclitaxel + Lapatinib + Trastuzumab	51.3%	difference 21.1%; 95% CI 9.1-34.2; *P* = 0.0001).
		ALLTO	NCT00490139	Phase III	Adjuvant	DFS	Trastuzumab	86.0%	
							Trastuzumab + Lapatinib	88.0%	
							Sequential Trastuzumab/Lapatinib		
		Geyer and al.	NCT00078572	Phase III	Locally advanced/MBC	TTP	Capecitabine + Lapatinib Capecitabine	8.4 months	HR 0.57; 95% CI 0.44-0.77; *P* <0.001
								4.4 month	
		GeparQuinto	NCT00567554	Phase III	Neo adjuvant	pCR	Chemotherapy + Trastuzumab	30.3%	OR 0 · 68; 95% CI 0.47-0.97; *P* = 0 · 04
							Chemotherapy + Lapatinib	22.7%	
Bevacizumab	VEGFA	AVEREL		Phase III	Locally recurrent/MBC	PFS	Docetaxel + Trastuzumab + Bevacizumab Docetaxel + Trastuzumab	16.8 months	
								13.9 months	
		BETH	NCT00625898	Phase III	Adjuvant	DFS	Chemotherapy + Trastuzumab + Bevacizumab Chemotherapy + Trastuzumab		HR 1.00; 95% CI 0.79–1.26
Everolimus	m TOR	BOLERO-3	NCT01007942	Phase III	Locally advanced/MBC	PFS	Trastuzumab + Vinorelbine + Everolimus Trastuzumab + Vinorelbine	30.4 weeks	HR 0.78; 95% CI 0.65-0.96; *P* = 0.0067
								25.1 weeks	

CI, confidence interval; DFS, disease free survival; HR, hazard ratio; MBC, metastatic breast cancer; mTOR, mammalian target of rapamycin; OR, odds ratio; pCR, pathological complete response; PFS, progression free survival; TTP, time to progression; VEGFA, vascular endothelial growth factor A.

### Small tyrosine kinase inhibitors: Lapatinib and neratinib

#### *Lapatinib*

Lapatinib is a small molecule, dual tyrosine kinase inhibitor (TKI) of EGFR and HER2. It inhibits the intracellular kinase domain of HER2 contrary to trastuzumab that inhibits the extracellular domain and blocks ligand-induced heterodimer signaling. It could prevent signaling related to truncated HER2 receptor or enhance trastuzumab-dependant ADCC, thanks to an accumulation of HER2 at the cell surface.

It has shown its efficacy when combined with capecitabine, in terms of time to progression (hazard ratio (HR): 0.57; 95% confidence interval (CI) 0.43 to 0.77; *P* <0.001). In the same trial, the median overall survival times were 75 weeks for the combination arm and 64.7 weeks for the capecitabine arm (HR 0.87; 95% CI 0.71 to 1.08; *P* = 0.210) [36–38]. Lapatinib has also shown its efficacy in terms of OS, when combined with paclitaxel, over paclitaxel single agent (HR 0.74; 95% CI 0.58 to 0.94; *P* = 0.0124) [39].

The efficacy of dual HER2 blockade with trastuzumab and lapatinib was investigated in the phase III study EGF104900. Patients with HER2-positive metastatic breast cancer (MBC) who progressed during trastuzumab treatment were randomly assigned to receive lapatinib in monotherapy or lapatinib in combination with trastuzumab. The combination was associated with better outcome both in progression free survival (PFS) (HR 0.74; 95% CI 0.58 to 0.94; *P* = 0.011) and OS (HR 0.74; 95% CI 0.57 to 0.97; *P* = 0.026) [40].

Following the results of these randomized trials in the metastatic setting, the efficacy of lapatinib has been investigated in early breast cancer, mainly in the context of trials testing dual inhibition of HER2. In the phase III trial of NSABP (protocol B-41), 529 patients were randomized to receive weekly paclitaxel with either trastuzumab weekly, lapatinib daily or the association trastuzumab plus lapatinib before undergoing surgery. Lapatinib alone had a similar percentage of pCR as trastuzumab (53.2% and 52.5%, respectively; *P* = 0.98). The dual HER2 blockade was associated with a higher pCR (62%; 95% CI 54.3 to 68.8) as compared to single-agent HER2 therapy but the difference was not statistically significant (*P* = 0.095) [41].

In another randomized trial performed in the neo-adjuvant setting (Neo ALTTO), patients were randomized among lapatinib, trastuzumab or lapatinib plus trastuzumab, all in combination with paclitaxel after six weeks of targeted therapy alone. The pCR rate was significantly higher in the group treated with dual inhibition (51.3%) as compared to trastuzumab alone (29.5%) (a 21.1% difference; 95% CI 9.1 to 34.2; *P* = 0.0001). There was no significant difference in pCR between the lapatinib and the trastuzumab group [42].

First results from the phase III ALLTO trial comparing one year of lapatinib alone, trastuzumab alone, their sequence or their combination in an adjuvant setting in 8,381 HER2 positive breast cancers have been reported [42]. According to a predefined statistical plan, there was no statistically significant difference between dual targeting HER2 and trastuzumab (four years DFS: 88% versus 86%; HR 0.84; 97.5% CI 0.70 to 1.02; *P* = 0.048). Nevertheless, this lack of benefit could be related to the low number of events.

### Neratinib

Neratinib is an oral irreversible pan-HER inhibitor. Preclinical data on breast cancer cell lines suggest that it could overcome both primary and acquired trastuzumab resistance in HER2 positive breast cancer cell lines [43].

Neratinib has shown antitumor activity among both pretreated and trastuzumab naive patients. In a phase II trial, the median PFS observed with neratinib was 22.3 weeks among patients with prior trastuzumab treatment and 39.6 weeks with trastuzumab naive patients. Objective response rates were 24% and 56%, respectively [44].

Neratinib single agent has been compared with the association of lapatinib plus capecitabine. Both median PFS (4.5 months) and OS (19.7 months) for neratinib single agent were found to be numerically inferior to that of the combination therapy, although not statistically significant (PFS 4.5 months versus 6.8 months; OS 19.7 months versus 23.6 months, *P* = 0.231 and *P* = 0.280, respectively) [45].

Neratinib is currently developed in combination with paclitaxel, vinorelbine, capecitabine and temsirolimus [46–49]. The association of neratinib and capecitabine has been studied in a phase I/II trial. The median PFS was 40.3 weeks and the overall response rate was 57% for patients with no prior lapatinib treatment [50]. An ongoing phase III study (NCT00915018) compares weekly paclitaxel with either neratinib or trastuzumab as first-line treatment for HER2-positive MBC.

The most common adverse event of this treatment is diarrhea in more than 90% of the cases, and then neutropenia (50% of the cases) [46,48].

### Vascular endothelial growth factor inhibitors

The vascular endothelial growth factor (VEGF) plays a key role in progression of this cancer by promoting tumor angiogenesis [51]. Bevacizumab is a humanized monoclonal antibody which targets VEGF and inhibits VEGF signaling pathways.

In the AVEREL study, the efficacy of bevacizumab was evaluated in first line therapy for locally recurrent or metastatic HER2 positive breast cancer. In this phase III study, the HR for progression was 0.72 (95% CI 0.54 to 0.94; *P* = 0.0162). The median PFS was 16.8 months in the bevacizumab arm versus 13.9 months [52].

Bevacizumab was also evaluated in HER2 positive, early breast cancer (BETH trial). In this trial, patients were randomly assigned to receive chemotherapy, trastuzumab plus bevacizumab, or chemotherapy and trastuzumab alone. No difference of efficacy was observed between the two arms (DFS, HR 1.00; 95% CI 0.79 to 1.26) [53].

Other antiangiogenic agents that can target VEGFR, such as multitargeted anti-angiogenic TKIs (sunitinib, sorafenib, vandetanib) have shown promising results [54].

### Monoclonal antibodies: pertuzumab

Resistance to trastuzumab can be explained by signaling through other HER dimerization [26]. Pertuzumab is an anti-HER2 antibody that inhibits HER2/HER3 dimerizations [26,55] and, thus, can prevent these potential escape pathways. Binding of trastuzumab and pertuzumab is not mutually exclusive and their differing mechanisms of action act complementarily to provide a more complete blockade of HER2 signal transduction. The combination treatment with trastuzumab and pertuzumab has shown strongly enhanced antitumor activity in xenograft models with reduction in the levels of HER proteins [56].

Cortes *et al*. have evaluated whether adding pertuzumab to trastuzumab could reverse trastuzumab resistance. The objective response rate and clinical benefit rate (CBR) were 3.4% and 10.3% in patients who received pertuzumab monotherapy after trastuzumab. At the opposite end, objective response rates and CBR were 17.6% and 41.2% in patients who received the combination after progression on trastuzumab [57].

Based on these data, the efficacy of adding pertuzumab to trastuzumab has been investigated.

In the CLEOPATRA study, patients were randomized between placebo plus trastuzumab plus docetaxel or pertuzumab plus trastuzumab plus docetaxel. The median PFS was 12.4 months in the placebo group versus 18.7 months in the pertuzumab group (HR 0.69; 95% CI 0.58 to 0.81; *P* <0.001). The pertuzumab arm was also associated with an OS improvement. The median OS in the placebo group was 37.6 months (95% CI 34.3 to NE (not estimable)) and had been not reached in the pertuzumab group (95% CI 42.4 to NE) (HR 0.66; 95% CI 0.52 to 0.84; *P* = 0.0008) [58,59].

The combination of chemotherapy with trastuzumab and pertuzumab has also shown interesting results in the neo-adjuvant setting.

The NeoSphere study (multicenter phase II study) evaluated the efficacy of dual inhibition using pertuzumab. Patients who received pertuzumab and trastuzumab plus docetaxel had a significantly higher pCR rate (45.8%; 95% CI 36.1 to 55.7) compared to patients treated with trastuzumab and docetaxel (29%; 95% CI 20.6 to 38.5; *P* = 0.0141). Interestingly, pertuzumab and trastuzumab without chemotherapy was associated with a 16.8% pCR [14].

TRYPHAENA is another comparative trial performed in the neoadjuvant setting. This trial investigated the efficacy of trastuzumab and pertuzumab with three cycles of FEC (5-fluorouracil, epirubicine, cyclophosphamide) then three cycles of docetaxel, or three FEC then three docetaxel with the combination of trastuzumab and pertuzumab or docetaxel plus carboplatin plus the combination during six cycles. The primary endpoint was to assess the cardiac safety. The pCR rates were quite similar in the three arms (61.6%, 57%, and 66.2%, respectively) [60].

In the early stage of breast cancer, the efficacy of pertuzumab is currently being investigated in the adjuvant setting (APHINITY trial).

### Antibody-drug conjugates: T-DM1

Trastuzumab emtansine (T-DM1) is an antibody-drug conjugate and is composed of trastuzumab covalently linked to maytansine, a cytotoxic agent [61].

The EMILIA study, a phase III registration trial compared T-DM1 to lapatinib and capecitabine in patients with HER2 positive advanced breast cancer previously treated with trastuzumab and a taxane. The median PFS was 9.6 months with T-DM1 versus 6.4 months with lapatinib plus capecitabine (HR 0.65; 95% CI 0.55 to 0.77; *P* <0.001). The median OS at the second interim analysis was 30.9 months in the T-DM1 arm versus 25.1 months in the lapatinib arm (HR 0.68; 95% CI 0.55 to 0.85; *P* <0.001) [62].

The phase III TH3RESA trial compared third line treatment (including trastuzumab and lapatinib) of metastatic or unresectable locally advanced or recurrent HER2 positive breast cancer with T-DM1 to the treatment of the physician’s choice. T-DM1 treatment significantly improved PFS compared with physician’s choice (median PFS 6.2 months versus 3.3 months; HR 0.528; 95% CI 0.422 to 0.661; *P* <0.0001). Final overall survival analysis is still awaited but interim analysis showed a trend favoring T-DM1 with a lower incidence of grade 3 or worse adverse events [63].

Another ongoing phase III trial, MARIANNE (NCT01120184), compares single-agent T-DM1 to T-DM1 combined with pertuzumab to trastuzumab plus a taxane in first line treatment of metastatic breast cancer.

These studies will provide more information about the indications of T-DM1 in the treatment algorithms for HER2-positive disease.

### mTOR inhibitors/PI3K inhibitors

The mammalian target of rapamycin (mTOR) is a serine-threonine protein kinase that mediates mRNA translation and protein synthesis. Activation of this pathway is known as a mechanism of trastuzumab resistance [29,64]. Preclinical studies have suggested that mTOR targeting could reverse resistance to trastuzumab [65].

In a phase I/II study, patients with HER2-positive metastatic breast cancer received trastuzumab combined with everolimus, after resistance to trastuzumab. Fifteen percent of patients had a partial response and 19% had a long stable disease (≥6 months). The clinical benefit rate was 34% [66].

A phase II study evaluated the efficacy of everolimus combined with trastuzumab and paclitaxel in patients who were resistant to trastuzumab and taxane therapy. The median PFS was 5.5 months and the median OS was 18.1 months [67]. This combination is currently being evaluated in the BOLERO-1 trial.

The BOLERO-3 study compared the combination of everolimus, trastuzumab plus vinorelbine to trastuzumab and vinorelbine. The association of the mTOR inhibitor with vinorelbine significantly improved PFS (30.4 weeks in the everolimus arm versus 25.1 weeks in the placebo arm; HR 0.78; 95% CI 0.65 to 0.96; *P* = 0.0067). In this study, several biomarkers (PTEN, PIK3CA and pS6) were analyzed to find some subpopulation for whom the benefit of everolimus was higher. Patients with a low PTEN and high pS6 level seemed to derive more benefit from addition of everolimus. Median PFS gain was 12 weeks for the high pS6 level subgroup (HR 0.48; 95% CI 0.24 to 0.96) and 18 weeks for the low PTEN subgroup (HR 0.41; 95% CI 0.20 to 0.82). Unfortunately, there was no marker-treatment interaction with PIK3CA mutation. These promising results deserve additional research.

Building on these results, the combination between trastuzumab and BKM120, an oral pan-class 1 PI3K inhibitor, has been developed. It inhibits this signaling pathway thanks to an ATP-competitive action. Several objective responses were observed in a phase I study [68], and this combination is currently being investigated in a randomized trial in the neoadjuvant setting (neoPHOEBE trial).

### HSP90 inhibitors

Heat shock protein 90 (HSP90) is a chaperone which stabilizes oncogenic proteins. Inhibition of HSP90 leads to the degradation of these proteins involved in cancer biology [69]. HSP90 inhibitors are currently being developed in ALK-translocated lung cancer, myeloma and gastrointestinal stromal tumor (GIST). In HER2 positive breast cancer, HSP90 inhibitors have shown antitumor activity when combined with trastuzumab [70].

17-Demethoxygeldanamycin (17-AAG) inhibits the activity of HSP90, thereby inducing the degradation of many oncogenic proteins. In a phase II study, 17-AAG (tanespimycin) was given in combination with trastuzumab in patients who previously failed to trastuzumab. The overall response rate (ORR) was 22%, the CBR was 59%, the median PFS was six months and the median OS was seventeen months [71].

In another phase II study, retaspimycin (IPI-504) given with trastuzumab showed modest clinical activity, but it is possible that under-dosing limited efficacy [72]. Other studies employing higher doses are ongoing.

These findings are promising and other studies are expected to develop these new targeted therapies.

### Blockade of PD-1/PD-L1 immune checkpoint

Programmed death 1 (PD-1) is a co-inhibitory receptor and acts as a negative regulator of the immune system. It is overexpressed on tumor-infiltrating lymphocytes (TIL). The PD-1 ligand, PD-L1, is expressed by multiple carcinoma, including breast cancers. This suggests that the PD-1/PD-L1 signaling pathway could be a candidate target in breast and other cancers.

T cell infiltration is predictive for the efficacy of trastuzumab [73–76]. Biomarker studies have shown that PD-1+ TILs are associated with poor prognosis in HER2 positive breast cancer [77,78] and preliminary data also suggest a relationship between PD-L1 expression on tumor cells and objective response to anti PD-1 therapy [79]. The effects of anti-PD-1 and anti-PD-L1 antibodies have been investigated in phase I trials in various cancer types and have shown encouraging responses (response rate 6 to 28% and stabilized disease rate 12% to 41%) [80,81].

Preclinical studies have shown a synergism between trastuzumab and anti-PD1 antibodies [82].

Other monoclonal antibody (mAb)-based therapies are being investigated including anti CD73. Pre-clinical data have suggested that it can delay tumor growth and inhibit the development of metastases [83].

## Conclusions

Although trastuzumab remains the standard treatment in patients with HER2 overexpressing breast cancer in neoadjuvant, adjuvant and metastatic settings, the presence of acquired and *de novo* resistance is a serious concern. The understanding of resistance mechanisms could allow developing strategies to prevent or overcome this resistance. The development of novel targeted therapies has changed the practices in metastatic settings.

New standards of care include trastuzumab plus pertuzumab plus docetaxel in first line treatment and TDM-1 for trastuzumab-resistant patients. In early breast cancer, dual HER2 blockade has shown promising results in the neoadjuvant setting. This strategy is being evaluated in the adjuvant setting in several randomized trials.

Since several different targets are under investigation, there is a need to identify predictive biomarkers to optimize combination strategies for suitable patients. Loss of PTEN and a high level of pS6 could facilitate the selection of appropriate patients who can benefit from personalized targeted therapy.

## Abbreviations

17-AAG: 17-demethoxygeldanamycine
95% CI: confidence interval at 95%
ADCC: antibody-dependent cell-mediated cytotoxicity
CBR: clinical benefit rate
DFS: disease free survival
EGFR: epidermal growth factor receptor
HER2: human epidermal growth factor receptor 2
HR: hazard ratio
HSP90: heat shock protein 90
mAb: monoclonal antibody
MAPK: mitogen-activated protein kinase
MBC: metastatic breast cancer
mTOR: mammalian target of rapamycin
ORR: overall response rate
OS: overall survival
pCR: pathological complete response
PD-1: programmed death 1
PD-L1: programmed death 1 ligand
PFS: progression free survival
PI3K: phosphoinositide 3-kinase
PTEN: phosphatase and tensin homolog
T-DM1: trastuzumab emtansine
TIL: tumor-infiltrating lymphocytes
TKI: tyrosine kinase inhibitor
VEGF: vascular endothelial growth factor

## References

[1] Slamon DJ, Clark GM (1988). Amplification of c-erbB-2 and aggressive human breast tumors?. Science.

[2] Ravdin PM, Chamness GC (1995). The c-erbB-2 proto-oncogene as a prognostic and predictive marker in breast cancer: a paradigm for the development of other macromolecular markers–a review. Gene.

[3] Pegram M, Slamon D (2000). Biological rationale for HER2/neu (c-erbB2) as a target for monoclonal antibody therapy. Semin Oncol.

[4] Sjögren S, Inganäs M, Lindgren A, Holmberg L, Bergh J (1998). Prognostic and predictive value of c-erbB-2 overexpression in primary breast cancer, alone and in combination with other prognostic markers. J Clin Oncol.

[5] Ross JS, Fletcher JA (1998). The HER-2/neu oncogene in breast cancer: prognostic factor, predictive factor, and target for therapy. Stem Cells.

[6] Hamilton A, Piccart M (2000). The contribution of molecular markers to the prediction of response in the treatment of breast cancer: a review of the literature on HER-2, p53 and BCL-2. Ann Oncol.

[7] Gennari A, Sormani MP, Pronzato P, Puntoni M, Colozza M, Pfeffer U, Bruzzi P (2008). HER2 status and efficacy of adjuvant anthracyclines in early breast cancer: a pooled analysis of randomized trials. J Natl Cancer Inst.

[8] Houston SJ, Plunkett TA, Barnes DM, Smith P, Rubens RD, Miles DW (1999). Overexpression of c-erbB2 is an independent marker of resistance to endocrine therapy in advanced breast cancer. Br J Cancer.

[9] Borg A, Baldetorp B, Fernö M, Killander D, Olsson H, Rydén S, Sigurdsson H (1994). ERBB2 amplification is associated with tamoxifen resistance in steroid-receptor positive breast cancer. Cancer Lett.

[10] Musolino A, Ciccolallo L, Panebianco M, Fontana E, Zanoni D, Bozzetti C, Michiara M, Silini EM, Ardizzoni A (2011). Multifactorial central nervous system recurrence susceptibility in patients with HER2-positive breast cancer: epidemiological and clinical data from a population-based cancer registry study. Cancer.

[11] Slamon DJ, Leyland-Jones B, Shak S, Fuchs H, Paton V, Bajamonde A, Fleming T, Eiermann W, Wolter J, Pegram M, Baselga J, Norton L (2001). Use of chemotherapy plus a monoclonal antibody against HER2 for metastatic breast cancer that overexpresses HER2. N Engl J Med.

[12] Vogel CL, Cobleigh MA, Tripathy D, Gutheil JC, Harris LN, Fehrenbacher L, Slamon DJ, Murphy M, Novotny WF, Burchmore M, Shak S, Stewart SJ, Press M (2002). Efficacy and safety of trastuzumab as a single agent in first-line treatment of HER2-overexpressing metastatic breast cancer. J Clin Oncol.

[13] Eiermann W, International Herceptin Study Group (2001). Trastuzumab combined with chemotherapy for the treatment of HER2-positive metastatic breast cancer: pivotal trial data. Ann Oncol.

[14] Gianni L, Pienkowski T, Im YH, Roman L, Tseng LM, Liu MC, Lluch A, Staroslawska E, de la Haba-Rodriguez J, Im SA, Pedrini JL, Poirier B, Morandi P, Semiglazov V, Srimuninnimit V, Bianchi G, Szado T, Ratnayake J, Ross G, Valagussa P (2012). Efficacy and safety of neoadjuvant pertuzumab and trastuzumab in women with locally advanced, inflammatory, or early HER2-positive breast cancer (NeoSphere): a randomised multicentre, open-label, phase 2 trial. Lancet Oncol.

[15] Dahabreh IJ, Linardou H, Siannis F, Fountzilas G, Murray S (2008). Trastuzumab in the adjuvant treatment of early-stage breast cancer: a systematic review and meta-analysis of randomized controlled trials. Oncologist.

[16] Piccart-Gebhart MJ, Procter M, Leyland-Jones B, Goldhirsch A, Untch M, Smith I, Gianni L, Baselga J, Bell R, Jackisch C, Cameron D, Dowsett M, Barrios CH, Steger G, Huang CS, Andersson M, Inbar M, Lichinitser M, Láng I, Nitz U, Iwata H, Thomssen C, Lohrisch C, Suter TM, Rüschoff J, Suto T, Greatorex V, Ward C, Straehle C, McFadden E (2005). Trastuzumab after adjuvant chemotherapy in HER2-positive breast cancer. N Engl J Med.

[17] Buzdar AU, Ibrahim NK, Francis D, Booser DJ, Thomas ES, Theriault RL, Pusztai L, Green MC, Arun BK, Giordano SH, Cristofanilli M, Frye DK, Smith TL, Hunt KK, Singletary SE, Sahin AA, Ewer MS, Buchholz TA, Berry D, Hortobagyi GN (2005). Significantly higher pathologic complete remission rate after neoadjuvant therapy with trastuzumab, paclitaxel, and epirubicin chemotherapy: results of a randomized trial in human epidermal growth factor receptor 2-positive operable breast cancer. J Clin Oncol.

[18] Untch M, Rezai M, Loibl S, Fasching PA, Huober J, Tesch H, Bauerfeind I, Hilfrich J, Eidtmann H, Gerber B, Hanusch C, Kühn T, du Bois A, Blohmer JU, Thomssen C, Dan Costa S, Jackisch C, Kaufmann M, Mehta K, von Minckwitz G (2010). Neoadjuvant treatment with trastuzumab in HER2-positive breast cancer: results from the GeparQuattro study. J Clin Oncol.

[19] Musolino A, Naldi N, Bortesi B, Pezzuolo D, Capelletti M, Missale G, Laccabue D, Zerbini A, Camisa R, Bisagni G, Neri TM, Ardizzoni A (2008). Immunoglobulin G fragment C receptor polymorphisms and clinical efficacy of trastuzumab-based therapy in patients with HER-2/neu-positive metastatic breast cancer. J Clin Oncol.

[20] Yakes FM, Chinratanalab W, Ritter CA, King W, Seelig S, Arteaga CL (2002). Herceptin-induced inhibition of phosphatidylinositol-3 kinase and Akt Is required for antibody-mediated effects on p27, cyclin D1, and antitumor action. Cancer Res.

[21] Lu Y, Zi X, Zhao Y, Pollak M (2004). Overexpression of ErbB2 receptor inhibits IGF-I-induced Shc-MAPK signaling pathway in breast cancer cells. Biochem Biophys Res Commun.

[22] Dubská L, Andera L, Sheard MA (2005). HER2 signaling downregulation by trastuzumab and suppression of the PI3K/Akt pathway: an unexpected effect on TRAIL-induced apoptosis. FEBS Lett.

[23] Romond EH, Perez EA, Bryant J, Suman VJ, Geyer CE, Davidson NE, Tan-Chiu E, Martino S, Paik S, Kaufman PA, Swain SM, Pisansky TM, Fehrenbacher L, Kutteh LA, Vogel VG, Visscher DW, Yothers G, Jenkins RB, Brown AM, Dakhil SR, Mamounas EP, Lingle WL, Klein PM, Ingle JN, Wolmark N (2005). Trastuzumab plus adjuvant chemotherapy for operable HER2-positive breast cancer. N Engl J Med.

[24] Garrett JT, Arteaga CL (2011). Resistance to HER2-directed antibodies and tyrosine kinase inhibitors. Cancer Biol Ther.

[25] Liu B, Fan Z, Edgerton SM, Yang X, Lind SE, Thor AD (2011). Potent anti-proliferative effects of metformin on trastuzumab-resistant breast cancer cells via inhibition of erbB2/IGF-1 receptor interactions. Cell Cycle.

[26] Nahta R (2012). Pharmacological strategies to overcome HER2 cross-talk and Trastuzumab resistance. Curr Med Chem.

[27] Saal LH, Holm K, Maurer M, Memeo L, Su T, Wang X, Yu JS, Malmström PO, Mansukhani M, Enoksson J, Hibshoosh H, Borg A, Parsons R (2005). PIK3CA mutations correlate with hormone receptors, node metastasis, and ERBB2, and are mutually exclusive with PTEN loss in human breast carcinoma. Cancer Res.

[28] Shattuck DL, Miller JK, Carraway KL, Sweeney C (2008). Met receptor contributes to trastuzumab resistance of Her2-overexpressing breast cancer cells. Cancer Res.

[29] Pandolfi PP (2004). Breast cancer — loss of PTEN predicts resistance to treatment. N Engl J Med.

[30] Rexer BN, Arteaga CL (2012). Intrinsic and acquired resistance to HER2-targeted therapies in HER2 gene-amplified breast cancer: mechanisms and clinical implications. Crit Rev Oncog.

[31] Mukhopadhyay P, Chakraborty S, Ponnusamy MP, Lakshmanan I, Jain M, Batra SK (2011). Mucins in the pathogenesis of breast cancer: implications in diagnosis, prognosis and therapy. Biochim Biophys Acta.

[32] Mukohara T (2011). Mechanisms of resistance to anti-human epidermal growth factor receptor 2 agents in breast cancer. Cancer Sci.

[33] Yang W, Klos K, Yang Y, Smith TL, Shi D, Yu D (2002). ErbB2 overexpression correlates with increased expression of vascular endothelial growth factors A, C, and D in human breast carcinoma. Cancer.

[34] Tural D, Serdengecti S, Demirelli F, Oztürk T, Ilvan S, Turna H, Ozgüroglu M, Büyükünal E (2014). Clinical significance of p95HER2 overexpression, PTEN loss and PI3K expression in p185HER2-positive metastatic breast cancer patients treated with trastuzumab-based therapies. Br J Cancer.

[35] Gallardo A, Lerma E, Escuin D, Tibau A, Muñoz J, Ojeda B, Barnadas A, Adrover E, Sánchez-Tejada L, Giner D, Ortiz-Martínez F, Peiró G (2012). Increased signalling of EGFR and IGF1R, and deregulation of PTEN/PI3K/Akt pathway are related with trastuzumab resistance in HER2 breast carcinomas. Br J Cancer.

[36] Geyer CE, Forster J, Lindquist D, Chan S, Romieu CG, Pienkowski T, Jagiello-Gruszfeld A, Crown J, Chan A, Kaufman B, Skarlos D, Campone M, Davidson N, Berger M, Oliva C, Rubin SD, Stein S, Cameron D (2006). Lapatinib plus capecitabine for HER2-positive advanced breast cancer. N Engl J Med.

[37] Cameron D, Casey M, Press M, Lindquist D, Pienkowski T, Romieu CG, Chan S, Jagiello-Gruszfeld A, Kaufman B, Crown J, Chan A, Campone M, Viens P, Davidson N, Gorbounova V, Raats JI, Skarlos D, Newstat B, Roychowdhury D, Paoletti P, Oliva C, Rubin S, Stein S, Geyer CE (2008). A phase III randomized comparison of lapatinib plus capecitabine versus capecitabine alone in women with advanced breast cancer that has progressed on trastuzumab: updated efficacy and biomarker analyses. Breast Cancer Res Treat.

[38] Cameron D, Casey M, Oliva C, Newstat B, Imwalle B, Geyer CE (2010). Lapatinib plus capecitabine in women with HER-2-positive advanced breast cancer: final survival analysis of a phase III randomized trial. Oncologist.

[39] Guan Z, Xu B, DeSilvio ML, Shen Z, Arpornwirat W, Tong Z, Lorvidhaya V, Jiang Z, Yang J, Makhson A, Leung WL, Russo MW, Newstat B, Wang L, Chen G, Oliva C, Gomez H (2013). Randomized trial of lapatinib versus placebo added to paclitaxel in the treatment of human epidermal growth factor receptor 2-overexpressing metastatic breast cancer. J Clin Oncol.

[40] Blackwell KL, Burstein HJ, Storniolo AM, Rugo HS, Sledge G, Aktan G, Ellis C, Florance A, Vukelja S, Bischoff J, Baselga J, O’Shaughnessy J (2012). Overall survival benefit with lapatinib in combination with trastuzumab for patients with human epidermal growth factor receptor 2-positive metastatic breast cancer: final results from the EGF104900 Study. J Clin Oncol.

[41] Robidoux A, Tang G, Rastogi P, Geyer CE, Azar CA, Atkins JN, Fehrenbacher L, Bear HD, Baez-Diaz L, Sarwar S, Margolese RG, Farrar WB, Brufsky AM, Shibata HR, Bandos H, Paik S, Costantino JP, Swain SM, Mamounas EP, Wolmark N (2013). Lapatinib as a component of neoadjuvant therapy for HER2-positive operable breast cancer (NSABP protocol B-41): an open-label, randomised phase 3 trial. Lancet Oncol.

[42] Baselga J, Bradbury I, Eidtmann H, Di Cosimo S, de Azambuja E, Aura C, Gómez H, Dinh P, Fauria K, Van Dooren V, Aktan G, Goldhirsch A, Chang TW, Horváth Z, Coccia-Portugal M, Domont J, Tseng LM, Kunz G, Sohn JH, Semiglazov V, Lerzo G, Palacova M, Probachai V, Pusztai L, Untch M, Gelber RD, Piccart-Gebhart M, NeoALTTO Study Team (2012). Lapatinib with trastuzumab for HER2-positive early breast cancer (NeoALTTO): a randomised, open-label, multicentre, phase 3 trial. Lancet.

[43] Canonici A, Gijsen M, Mullooly M, Bennett R, Bouguern N, Pedersen K, O’Brien NA, Roxanis I, Li JL, Bridge E, Finn R, Siamon D, McGowan P, Duffy MJ, O’Donovan N, Crown J, Kong A (2013). Neratinib overcomes trastuzumab resistance in HER2 amplified breast cancer. Oncotarget.

[44] Burstein HJ, Sun Y, Dirix LY, Jiang Z, Paridaens R, Tan AR, Awada A, Ranade A, Jiao S, Schwartz G, Abbas R, Powell C, Turnbull K, Vermette J, Zacharchuk C, Badwe R (2010). Neratinib, an irreversible ErbB receptor tyrosine kinase inhibitor, in patients with advanced ErbB2-positive breast cancer. J Clin Oncol.

[45] Martin M, Bonneterre J, Geyer CE, Ito Y, Ro J, Lang I, Kim SB, Germa C, Vermette J, Wang K, Wang K, Awada A (2013). A phase two randomised trial of neratinib monotherapy versus lapatinib plus capecitabine combination therapy in patients with HER2+ advanced breast cancer. Eur J Cancer.

[46] Chow LW, Xu B, Gupta S, Freyman A, Zhao Y, Abbas R, Van Vo ML, Bondarenko I (2013). Combination neratinib (HKI-272) and paclitaxel therapy in patients with HER2-positive metastatic breast cancer. Br J Cancer.

[47] Jankowitz RC, Abraham J, Tan AR, Limentani SA, Tierno MB, Adamson LM, Buyse M, Wolmark N, Jacobs SA (2013). Safety and efficacy of neratinib in combination with weekly paclitaxel and trastuzumab in women with metastatic HER2-positive breast cancer: an NSABP Foundation Research Program phase I study. Cancer Chemother Pharmacol.

[48] Awada A, Dirix L, Manso Sanchez L, Xu B, Luu T, Diéras V, Hershman DL, Agrapart V, Ananthakrishnan R, Staroslawska E (2013). Safety and efficacy of neratinib (HKI-272) plus vinorelbine in the treatment of patients with ErbB2-positive metastatic breast cancer pretreated with anti-HER2 therapy. Ann Oncol.

[49] Gandhi L, Bahleda R, Tolaney SM, Kwak EL, Cleary JM, Pandya SS, Hollebecque A, Abbas R, Ananthakrishnan R, Berkenblit A, Krygowski M, Liang Y, Turnbull KW, Shapiro GI, Soria JC (2014). Phase I study of neratinib in combination with temsirolimus in patients with human epidermal growth factor receptor 2-dependent and other solid tumors. J Clin Oncol.

[50] López-Tarruella S, Jerez Y, Márquez-Rodas I, Martín M (2012). Neratinib (HKI-272) in the treatment of breast cancer. Future Oncol.

[51] Hicklin DJ, Ellis LM (2005). Role of the vascular endothelial growth factor pathway in tumor growth and angiogenesis. J Clin Oncol.

[52] Gianni L, Romieu GH, Lichinitser M, Serrano SV, Mansutti M, Pivot X, Mariani P, Andre F, Chan A, Lipatov O, Chan S, Wardley A, Greil R, Moore N, Prot S, Pallaud C, Semiglazov V (2013). AVEREL: a randomized phase III Trial evaluating bevacizumab in combination with docetaxel and trastuzumab as first-line therapy for HER2-positive locally recurrent/metastatic breast cancer. J Clin Oncol.

[53] Baker H: **2013 San Antonio Breast Cancer Symposium.** *Lancet Oncol* 2014, **15:**138. 10.1016/s1470-2045(13)70594-1 24627905

[54] Wang Z, Wang M, Yang F, Nie W, Chen F, Xu J, Guan X (2014). Multitargeted antiangiogenic tyrosine kinase inhibitors combined to chemotherapy in metastatic breast cancer: a systematic review and meta-analysis. Eur J Clin Pharmacol.

[55] Cho HS, Mason K, Ramyar KX, Stanley AM, Gabelli SB, Denney DW, Leahy DJ (2003). Structure of the extracellular region of HER2 alone and in complex with the Herceptin Fab. Nature.

[56] Scheuer W, Friess T, Burtscher H, Bossenmaier B, Endl J, Hasmann M (2009). Strongly enhanced antitumor activity of trastuzumab and pertuzumab combination treatment on HER2-positive human xenograft tumor models. Cancer Res.

[57] Cortés J, Fumoleau P, Bianchi GV, Petrella TM, Gelmon K, Pivot X, Verma S, Albanell J, Conte P, Lluch A, Salvagni S, Servent V, Gianni L, Scaltriti M, Ross GA, Dixon J, Szado T, Baselga J (2012). Pertuzumab monotherapy after trastuzumab-based treatment and subsequent reintroduction of trastuzumab: activity and tolerability in patients with advanced human epidermal growth factor receptor 2-positive breast cancer. J Clin Oncol.

[58] Swain SM, Kim SB, Cortés J, Ro J, Semiglazov V, Campone M, Ciruelos E, Ferrero JM, Schneeweiss A, Knott A, Clark E, Ross G, Benyunes MC, Baselga J (2013). Pertuzumab, trastuzumab, and docetaxel for HER2-positive metastatic breast cancer (CLEOPATRA study): overall survival results from a randomised, double-blind, placebo-controlled, phase 3 study. Lancet Oncol.

[59] Baselga J, Cortés J, Kim SB, Im SA, Hegg R, Im YH, Roman L, Pedrini JL, Pienkowski T, Knott A, Clark E, Benyunes MC, Ross G, Swain SM, CLEOPATRA Study Group (2012). Pertuzumab plus trastuzumab plus docetaxel for metastatic breast cancer. N Engl J Med.

[60] Schneeweiss A, Chia S, Hickish T, Harvey V, Eniu A, Hegg R, Tausch C, Seo JH, Tsai YF, Ratnayake J, McNally V, Ross G, Cortés J (2013). Pertuzumab plus trastuzumab in combination with standard neoadjuvant anthracycline-containing and anthracycline-free chemotherapy regimens in patients with HER2-positive early breast cancer: a randomized phase II cardiac safety study (TRYPHAENA). Ann Oncol.

[61] Diéras V, Bachelot T (2014). The success story of trastuzumab emtansine, a targeted therapy in HER2-positive breast cancer. Target Oncol.

[62] Verma S, Miles D, Gianni L, Krop IE, Welslau M, Baselga J, Pegram M, Oh DY, Diéras V, Guardino E, Fang L, Lu MW, Olsen S, Blackwell K, EMILIA Study Group (2012). Trastuzumab emtansine for HER2-positive advanced breast cancer. N Engl J Med.

[63] Krop IE, Kim SB, González-Martín A, LoRusso PM, Ferrero JM, Smitt M, Yu R, Leung AC, Wildiers H, TH3RESA study collaborators (2014). Trastuzumab emtansine versus treatment of physician’s choice for pretreated HER2-positive advanced breast cancer (TH3RESA): a randomised, open-label, phase 3 trial. Lancet Oncol.

[64] Ma J, Meng Y, Kwiatkowski DJ, Chen X, Peng H, Sun Q, Zha X, Wang F, Wang Y, Jing Y, Zhang S, Chen R, Wang L, Wu E, Cai G, Malinowska-Kolodziej I, Liao Q, Liu Y, Zhao Y, Sun Q, Xu K, Dai J, Han J, Wu L, Zhao RC, Shen H, Zhang H (2010). Mammalian target of rapamycin regulates murine and human cell differentiation through STAT3/p63/Jagged/Notch cascade. J Clin Invest.

[65] Lu CH, Wyszomierski SL, Tseng LM, Sun MH, Lan KH, Neal CL, Mills GB, Hortobagyi GN, Esteva FJ, Yu D (2007). Preclinical testing of clinically applicable strategies for overcoming trastuzumab resistance caused by PTEN deficiency. Clin Cancer Res.

[66] Morrow PK, Wulf GM, Ensor J, Booser DJ, Moore JA, Flores PR, Xiong Y, Zhang S, Krop IE, Winer EP, Kindelberger DW, Coviello J, Sahin AA, Nuñez R, Hortobagyi GN, Yu D, Esteva FJ (2011). Phase I/II study of trastuzumab in combination with everolimus (RAD001) in patients with HER2-overexpressing metastatic breast cancer who progressed on trastuzumab-based therapy. J Clin Oncol.

[67] Hurvitz SA, Dalenc F, Campone M, O’Regan RM, Tjan-Heijnen VC, Gligorov J, Llombart A, Jhangiani H, Mirshahidi HR, Tan-Chiu E, Miao S, El-Hashimy M, Lincy J, Taran T, Soria JC, Sahmoud T, André F (2013). A phase 2 study of everolimus combined with trastuzumab and paclitaxel in patients with HER2-overexpressing advanced breast cancer that progressed during prior trastuzumab and taxane therapy. Breast Cancer Res Treat.

[68] Saura C, Bendell J, Jerusalem G, Su S, Ru Q, De Buck S, Mills D, Ruquet S, Bosch A, Urruticoechea A, Beck JT, Di Tomaso E, Sternberg DW, Massacesi C, Hirawat S, Dirix L, Baselga J (2014). Phase Ib study of Buparlisib plus Trastuzumab in patients with HER2-positive advanced or metastatic breast cancer that has progressed on Trastuzumab-based therapy. Clin Cancer Res.

[69] Conroy SE, Latchman DS (1996). Do heat shock proteins have a role in breast cancer?. Br J Cancer.

[70] Modi S, Stopeck AT, Gordon MS, Mendelson D, Solit DB, Bagatell R, Ma W, Wheler J, Rosen N, Norton L, Cropp GF, Johnson RG, Hannah AL, Hudis CA (2007). Combination of trastuzumab and tanespimycin (17-AAG, KOS-953) is safe and active in trastuzumab-refractory HER-2 overexpressing breast cancer: a phase I dose-escalation study. J Clin Oncol.

[71] Modi S, Stopeck A, Linden H, Solit D, Chandarlapaty S, Rosen N, D’Andrea G, Dickler M, Moynahan ME, Sugarman S, Ma W, Patil S, Norton L, Hannah AL, Hudis C (2011). HSP90 inhibition is effective in breast cancer: a phase II trial of tanespimycin (17-AAG) plus trastuzumab in patients with HER2-positive metastatic breast cancer progressing on trastuzumab. Clin Cancer Res.

[72] Modi S, Saura C, Henderson C, Lin NU, Mahtani R, Goddard J, Rodenas E, Hudis C, O’Shaughnessy J, Baselga J (2013). A multicenter trial evaluating retaspimycin HCL (IPI-504) plus trastuzumab in patients with advanced or metastatic HER2-positive breast cancer. Breast Cancer Res Treat.

[73] Denkert C, Loibl S, Noske A, Roller M, Müller BM, Komor M, Budczies J, Darb-Esfahani S, Kronenwett R, Hanusch C, von Törne C, Weichert W, Engels K, Solbach C, Schrader I, Dietel M, von Minckwitz G (2010). Tumor-associated lymphocytes as an independent predictor of response to neoadjuvant chemotherapy in breast cancer. J Clin Oncol.

[74] Issa-Nummer Y, Darb-Esfahani S, Loibl S, Kunz G, Nekljudova V, Schrader I, Sinn BV, Ulmer HU, Kronenwett R, Just M, Kühn T, Diebold K, Untch M, Holms F, Blohmer JU, Habeck JO, Dietel M, Overkamp F, Krabisch P, von Minckwitz G, Denkert C: **Prospective validation of immunological infiltrate for prediction of response to neoadjuvant chemotherapy in HER2-negative breast cancer–a substudy of the neoadjuvant GeparQuinto trial.** *PLoS One* 2013, **8:**e79775. 10.1371/journal.pone.0079775 PMC3846472 24312450

[75] Mahmoud S, Lee A, Ellis I, Green A (2012). CD8(+) T lymphocytes infiltrating breast cancer: a promising new prognostic marker?. Oncoimmunology.

[76] Loi S, Michiels S, Salgado R, Sirtaine N, Jose V, Fumagalli D, Kellokumpu-Lehtinen PL, Bono P, Kataja V, Desmedt C, Piccart MJ, Loibl S, Denkert C, Smyth MJ, Joensuu H, Sotiriou C (2014). Tumor infiltrating lymphocytes is prognostic and predictive for trastuzumab benefit in early breast cancer: results from the FinHER trial. Ann Oncol.

[77] Muenst S, Soysal SD, Gao F, Obermann EC, Oertli D, Gillanders WE (2013). The presence of programmed death 1 (PD-1)-positive tumor-infiltrating lymphocytes is associated with poor prognosis in human breast cancer. Breast Cancer Res Treat.

[78] Ghebeh H, Barhoush E, Tulbah A, Elkum N, Al-Tweigeri T, Dermime S: **FOXP3+ Tregs and B7-H1+/PD-1+ T lymphocytes co-infiltrate the tumor tissues of high-risk breast cancer patients: implication for immunotherapy.** *BMC Cancer* 2008, **8:**57. 10.1186/1471-2407-8-57 PMC2279136 18294387

[79] Topalian SL, Hodi FS, Brahmer JR, Gettinger SN, Smith DC, McDermott DF, Powderly JD, Carvajal RD, Sosman JA, Atkins MB, Leming PD, Spigel DR, Antonia SJ, Horn L, Drake CG, Pardoll DM, Chen L, Sharfman WH, Anders RA, Taube JM, McMiller TL, Xu H, Korman AJ, Jure-Kunkel M, Agrawal S, McDonald D, Kollia GD, Gupta A, Wigginton JM, Sznol M (2012). Safety, activity, and immune correlates of anti-PD-1 antibody in cancer. N Engl J Med.

[80] Brahmer JR, Tykodi SS, Chow LQ, Hwu WJ, Topalian SL, Hwu P, Drake CG, Camacho LH, Kauh J, Odunsi K, Pitot HC, Hamid O, Bhatia S, Martins R, Eaton K, Chen S, Salay TM, Alaparthy S, Grosso JF, Korman AJ, Parker SM, Agrawal S, Goldberg SM, Pardoll DM, Gupta A, Wigginton JM (2012). Safety and activity of anti–PD-L1 antibody in patients with advanced cancer. N Engl J Med.

[81] Brahmer JR, Drake CG, Wollner I, Powderly JD, Picus J, Sharfman WH, Stankevich E, Pons A, Salay TM, McMiller TL, Gilson MM, Wang C, Selby M, Taube JM, Anders R, Chen L, Korman AJ, Pardoll DM, Lowy I, Topalian SL (2010). Phase I study of single-agent anti-programmed death-1 (MDX-1106) in refractory solid tumors: safety, clinical activity, pharmacodynamics, and immunologic correlates. J Clin Oncol.

[82] Stagg J, Loi S, Divisekera U, Ngiow SF, Duret H, Yagita H, Teng MW, Smyth MJ (2011). Anti-ErbB-2 mAb therapy requires type I and II interferons and synergizes with anti-PD-1 or anti-CD137 mAb therapy. Proc Natl Acad Sci U S A.

[83] Stagg J, Divisekera U, McLaughlin N, Sharkey J, Pommey S, Denoyer D, Dwyer KM, Smyth MJ (2010). Anti-CD73 antibody therapy inhibits breast tumor growth and metastasis. Proc Natl Acad Sci U S A.

